# Social determinants of health and disparate disability accumulation in a cohort of Black, Hispanic, and White patients with multiple sclerosis

**DOI:** 10.1177/13524585231185046

**Published:** 2023-07-12

**Authors:** Christopher M Orlando, Carlos A Pérez, Paunel Agyei, Marwah Elsehety, Sonia Kaur Singh, Joseph Thomas, Omar Alaina, John A Lincoln

**Affiliations:** Division of Multiple Sclerosis and Neuroimmunology, Department of Neurology, McGovern Medical School, University of Texas Health Science Center at Houston, Houston, TX, USA; Maxine Mesinger Multiple Sclerosis Comprehensive Care Center, Department of Neurology, Baylor College of Medicine, Houston, TX, USA; Division of Multiple Sclerosis and Neuroimmunology, Department of Neurology, McGovern Medical School, University of Texas Health Science Center at Houston, Houston, TX, USA; Inova, Fairfax, VA, USA; Division of Multiple Sclerosis and Neuroimmunology, Department of Neurology, McGovern Medical School, University of Texas Health Science Center at Houston, Houston, TX, USA; Division of Multiple Sclerosis and Neuroimmunology, Department of Neurology, McGovern Medical School, University of Texas Health Science Center at Houston, Houston, TX, USA; Division of Multiple Sclerosis and Neuroimmunology, Department of Neurology, McGovern Medical School, University of Texas Health Science Center at Houston, Houston, TX, USA; Division of Multiple Sclerosis and Neuroimmunology, Department of Neurology, McGovern Medical School, University of Texas Health Science Center at Houston, Houston, TX, USA

**Keywords:** Multiple sclerosis, health disparity, minority and vulnerable populations, health equity, social determinants of health, neighborhood characteristics

## Abstract

**Background:**

Black and Hispanic patients with multiple sclerosis (MS) have been shown to accumulate greater multiple sclerosis–associated disability (MSAD) than White patients. Disparities in social determinants of health (SDOH) among these groups have also been reported.

**Objective:**

To determine the extent to which associations of race and ethnicity with MSAD may be attributable to differences in SDOH.

**Methods:**

Retrospective chart analysis of patients at an academic MS center grouped by self-identified Black (*n* = 95), Hispanic (*n* = 93), and White (*n* = 98) race/ethnicity. Individual patient addresses were geocoded and matched with neighborhood-level area deprivation index (ADI) and social vulnerability index (SVI).

**Results:**

Average Expanded Disability Status Scale (EDSS) scores at last-recorded evaluations of White patients (1.7 ± 2.0) were significantly lower than Black (2.8 ± 2.4, *p* = 0.001) and Hispanic (2.6 ± 2.6, *p* = 0.020) patients. Neither Black race nor Hispanic ethnicity was significantly associated with EDSS in multivariable linear regression models that included individual-level SDOH indicators and either ADI or SVI.

**Conclusion:**

Black race and Hispanic ethnicity are not significantly associated with EDSS in models that include individual and neighborhood-level SDOH indicators. Further research should elucidate mechanisms by which structural inequities affect MS disease course.

## Introduction

Differences in the frequency with which Black, Hispanic, and White persons are affected by MS and the severity thereof have been reported, but the causes of these differences remain unclear. The incidence of MS appears to be higher among Black persons and lower among Hispanic persons in comparison to White persons.^1,2^ More rapid disability accumulation among Black^3–7^ and Hispanic^6,8^ persons with multiple sclerosis (PwMS) compared to White PwMS has also been observed.

Social determinants of health (SDOHs) are the conditions in which people are born, grow, work, live, and age that influence health outcomes.^9^ Inequities in SDOH are thought to be chiefly responsible for tremendous racial and ethnic health disparities in the United States.^10,11^ There remains a paucity of research regarding the effect of SDOH in MS, but available data document socioeconomic inequities among racial and ethnic groups and suggest an influence on disparate outcomes.^12^ Black and Hispanic PwMS enrolled in the North American Research Committee on Multiple Sclerosis (NARCOMS) registry had lower incomes and were less likely to have private insurance compared to White PwMS.^13^ Another study of NARCOMS data found that Black PwMS had greater MS-associated disability (MSAD) compared to White PwMS, but the difference was attenuated by adjustment for socioeconomic status.^4^

Neighborhood-level indicators (NLI) of social conditions including publicly available census data are a useful tool in the study of SDOH. NLI have been used as surrogates for individual-level data since the two are highly correlated.^14^ In addition, they may reflect conditions within a patient’s neighborhood that affect health outcomes.^14^ Associations of neighborhood characteristics with MS symptom severity and physical activity have been reported.^15,16^

The present study utilizes regression modeling to determine the degree to which racial and ethnic differences in MSAD are attenuated by the inclusion of SDOH indicators, including NLI. Other studies^17–19^ have used a combination of individual-level data and NLI to show associations of MSAD with SDOH, but the work presented here is novel in that it specifically interrogates the relationships between SDOH and MSAD disparities.

## Methods

### Standard protocol approvals, registrations, and patient consents

Patient data were obtained under a human research and subject protocol approved by the University of Texas Health Science Center—McGovern Medical School institutional review board. Informed consent was waived because this was a database and chart review without direct patient contact.

### Study population

Prior publications describe the study cohort in detail.^20,21^ Briefly, three groups of 100 age- and sex-matched patients were created according to self-reported White, Black, and Hispanic race and ethnicity. Patients were seen at the MS Comprehensive Care Clinic at University of Texas Physicians group, a private tertiary referral clinic affiliated with McGovern Medical School, UTHealth. A diagnosis of MS was identified by ICD-9 (340) and/or ICD-10 (G35) codes, and individual records were subsequently reviewed to ensure that the diagnosis was clinically confirmed and patients met 2017 McDonald diagnostic criteria.^22^

### Data collection and neighborhood-level SDOH indices

Kurtzke Expanded Disability Status Scale^23^ (EDSS) scores were retrospectively tabulated from examinations documented in the electronic medical record (EMR) at the earliest- and last-recorded in-person clinical encounters using an algorithm previously described.^21^ Visit dates ranged from May 2009 to June 2022. Selected clinical and sociodemographic characteristics were also collected via retrospective EMR review. Comorbid conditions were identified by ICD-9 and ICD-10 codes, reported medical history, and documented medications.

Individual-level SDOH available in the EMR included education level, primary language, insurance status, and employment status.

Factor analysis has been used to generate several composite indices to facilitate the use of NLI in research. This study makes use of two such indices: the Social Vulnerability Index^24^ (SVI) and Area Deprivation Index^25,26^ (ADI). The census items included in each index are summarized in Table 1. Relationships with health outcomes have previously been documented for both ADI^25,27^ and SVI.^28,29^ SVI and ADI scores used were both generated from 2015 American Communities Survey data. SVI data (accessed at https://www.atsdr.cdc.gov/placeandhealth/svi/data_documentation_download.html) were available at the census tract level, each tract representing approximately 4000 people.^24^ SVI is reported as a percentile ranking determined by comparing each census tract to all tracts nationally. ADI data are available via the University of Wisconsin-Madison Neighborhood Atlas (https://www.neighborhoodatlas.medicine.wisc.edu) at the census block group level (approximately 1000 people).^26^ ADI scores reported here reflect the decile into which each block group falls when compared to all block groups in the same state as the address in question. The vast majority were in Texas.

**Table 1.** Census variables included in neighborhood SDOH composite indices.

Census variable	SVI^24^	ADI^25^
Per capita income	✓	
Median family income		✓
Percent of individuals below the poverty level	✓	
Percent of families below the poverty level		✓
Income disparity^a^		✓
Percent of population below 150% of the poverty threshold		✓
Percent of civilian labor force population aged 16 years or older unemployed	✓	✓
Percent of employed persons aged 16 years or older in white collar occupations		✓
Percent of population aged 25 years or older with <9 years of education		✓
Percent of population aged 25 years or older with less than a high school diploma	✓	✓
Percent of persons 65 years of age or older	✓	
Percent of persons age 17 years of age or younger	✓	
Percent of persons more than 5 years old with a disability	✓	
Percent of single-parent households with children under 18 years	✓	✓
Percent minority	✓	
Percent of persons 5 years of age or older who speak English “less than well”	✓	
Median home value		✓
Median gross rent		✓
Median monthly mortgage		✓
Percent of owner-occupied housing units		✓
Percent of housing structures with 10 or more units	✓	
Percent mobile homes	✓	
Percent households with more than one person per room	✓	✓
Percent households with no vehicle available	✓	✓
Percent of persons in group quarters	✓	
Percent of households without a telephone		✓
Percent of occupied housing units without complete plumbing		✓

SDOH = social determinants of health; SVI = Social Vulnerability Index; ADI = Area Deprivation Index.

a Defined as log of 100× the ratio of the number of households with <$10,000 in income to the number of households with $50,000 or more in income.^25^

Patient addresses at the time of earliest clinical encounter were matched with census tract and block group numbers using the United States Census Bureau geocoder (https://geocoding.geo.census.gov/geocoder). Though many patients relocated to new addresses between documented encounters, address information was not available at sufficiently regular time points to accurately track patients’ movement through different communities. The address at the earliest-recorded clinical encounter was used as the best available approximation of social conditions that could influence subsequent disease course. Geocoded addresses were matched with corresponding SVI and ADI scores.

### Statistical analyses

All statistical analysis was performed in Stata version 14.2 (StataCorp, College Station, TX, USA). Non-parametric statistical tests were used as all variables followed a non-parametric distribution as reflected by a composite measure of skewness and kurtosis calculated for each. Continuous variables were compared among the three groups using the Kruskall-Wallis H test. The three possible group pairings (Black vs. Hispanic, Black vs. White, and Hispanic vs. White) were also compared using the Wilcoxon Rank-Sum test. For categorical variables, chi-square analysis was used both for comparison of all three groups and the three pairings.

Linear regression was then used to model associations of EDSS at the last-recorded assessment with race, ethnicity, and various clinical and social determinants. Where applicable, categorical data were simplified to dichotomous dummy variables. In general, variables with *p* ⩽ 0.1 in univariable analysis were considered for inclusion in multivariable models. Care was taken to avoid erroneous exclusion of variables with associations whose statistical significance was only apparent in multivariable models (Simpson’s Paradox). Consideration was also given to potential confounding. For example, exposure to disease-modifying therapy (DMT) was excluded from the final models because it was associated with higher EDSS, suggesting a confounding relationship in which disease severity was a driver of both EDSS and DMT selection. Variables ultimately included in linear regression models of final EDSS score were Hispanic ethnicity (dichotomous), Black race (dichotomous), Male sex (dichotomous), age at final evaluation (continuous), diagnostic lag (continuous), primary progressive multiple sclerosis (PPMS) phenotype (dichotomous), history of tobacco use (dichotomous), comorbid hypertension (dichotomous), comorbid diabetes mellitus (dichotomous), employment status (dichotomous), private insurance (dichotomous), ADI state rank decile (continuous), and SVI percentile rank (continuous).

A total of six multivariable models were generated to examine the effect of including or excluding variables reflecting SDOH. The first model included no SDOH variables. The second included only individual-level SDOH indicators. The next two models also included ADI and SVI scores, respectively. These were compared against two additional models that did not include variables for race and ethnicity. Variance inflation factors were calculated for all models to evaluate for collinearity, and no values exceeded a pre-determined cutoff of 10. Adjusted *R*^2^ values were calculated to avoid inflation of *R*^2^ values by the inclusion of multiple variables.

## Results

**Figure 1.**
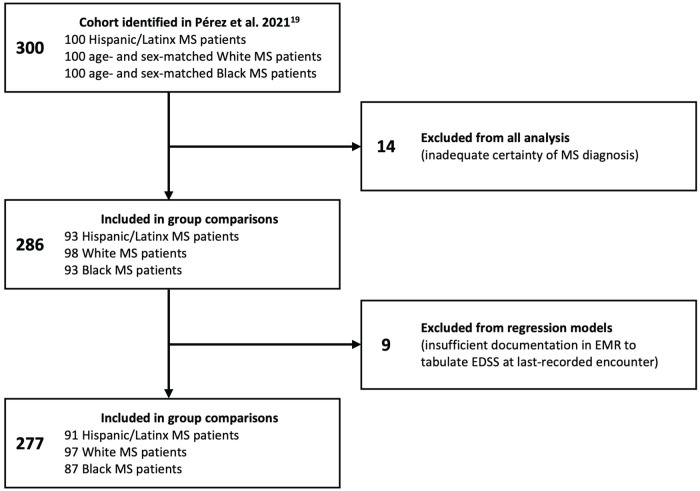
Flowchart of patient selection. MS: multiple sclerosis; EMR: electronic medical record; EDSS: Expanded Disability Status Scale.

A total of 14 PwMS were excluded from the previously reported cohort due to inadequate certainty of MS diagnosis. The remaining 286 patients included 95 Black patients, 93 Hispanic patients, and 98 White patients. Documentation of the earliest-available clinical visit was insufficient to tabulate an EDSS for seven patients, and the same was true of nine patients at the last-recorded clinical encounter. Patients without an EDSS at the latest-recorded encounter were excluded from the regression models, but other data from these patients were included in group comparisons (Figure 1). Data completeness was high for most remaining variables. Ten or more observations (3.5%) were missing for three variables: employment (22 missing), education level (65 missing), and vitamin D level (91 missing). Of these, only employment was included in multivariable regression models.

Table 2 summarizes clinical and sociodemographic characteristics of the three groups. Compared to White patients, average EDSS scores were higher among Black and Hispanic patients at both earliest- (*p* < 0.001) and last-recorded (*p* = 0.003) evaluations. There was no significant difference between the Black and Hispanic groups at either time point. The average difference between scores at the earliest- and last-recorded assessments did not differ significantly among the groups.

**Table 2.** Clinical and sociodemographic characteristics.

	Group			*p* Value			
	White (*n* = 98)	Black (*n* = 95)	Hispanic (*n* = 93)	Three-way comparison	Hispanic vs. White	Black vs White	Black vs Hispanic
Female sex, *n* (%)	76 (77.6)	75 (78.9)	73 (78.5)	0.972	0.875	0.814	0.940
Age at earliest evaluation, y, mean (SD)	33.8 (10.1)	35.3 (10.0)	32.4 (10.5)	0.161	0.309	0.312	0.063
Age at final evaluation, y, mean (SD)	41.8 (11.3)	42.0 (11.7)	39.8 (11.3)	0.391	0.273	0.841	0.207
Interval from earliest to final evaluation, y, mean (SD)	9.1 (12.6)	6.5 (4.9)	7.3 (4.4)	0.084	0.439	0.036	0.117
Age at symptom onset, y, mean (SD)	29.2 (9.5)	29.7 (8.9)	27.6 (9.8)	0.189	0.199	0.509	0.086
Diagnostic lag, y, mean (SD)	2.0 (3.2)	1.8 (3.8)	1.8 (3.3)	0.302	0.628	0.131	0.302
Disease phenotype				0.094	0.471	0.219	**0.050**
RRMS, *n* (%)	84 (85.7)	75 (78.9)	77 (82.8)				
SPMS *n* (%)	10 (10.2)	10 (10.5)	14 (15.1)				
PPMS, *n* (%)	4 (4.1)	10 (10.5)	2 (2.2)	0.032	0.444	0.084	0.019
Age at progression to SPMS, y, mean (SD)	42.0 (9.5)	39.7 (6.2)	39.1 (9.6)	0.776	0.395	0.790	0.953
Duration of DMT							
Total, y, mean (SD)	8.5 (5.7)	8.1 (5.8)	7.7 (5.2)	0.659	0.379	0.496	0.902
Highly efficacious, y, mean (SD)	1.7 (2.6)	1.7 (2.7)	2.3 (3.0)	0.184	0.102	0.898	0.077
Tobacco use							
Never smoker, *n* (%)	70 (71.4)	73 (76.8)	69 (74.2)	0.692	0.668	0.391	0.673
Former smoker, *n* (%)	19 (19.4)	11 (11.6)	18 (19.4)				
Current smoker, *n* (%)	9 (9.2)	11 (11.6)	6 (6.5)	0.473	0.483	0.585	0.220
Vitamin D at baseline, ng/mL, mean (SD)	42.1 (22.9)	26.4 (18.2)	36.4 (24.5)	< 0.001	0.218	< 0.001	0.015
Vitamin D deficiency (⩽ 20 ng/mL), *n* (%)	7 (7.1)	29 (30.5)	13 (14.0)	< 0.001	0.123	< 0.001	0.006
Vascular risk factors/comorbidities							
Obesity (BMI ⩾ 30), *n* (%)	27 (27.6)	40 (42.1)	30 (32.3)	0.094	0.477	0.034	0.163
Hypertension, *n* (%)	28 (28.6)	13 (13.7)	29 (31.2)	0.010	0.693	0.011	0.004
Hyperlipidemia, *n* (%)	7 (7.1)	4 (4.2)	9 (9.7)	0.339	0.527	0.380	0.140
Diabetes mellitus, *n* (%)	4 (4.1)	4 (4.2)	6 (6.5)	0.698	0.462	0.964	0.494
Educational attainment							
Less than 12 years, *n* (%)	10 (10.2)	10 (10.5)	21 (22.6)	0.028	0.040	0.819	0.019
Some college, *n* (%)	21 (21.4)	17 (17.9)	10 (10.8)				
Completed college, *n* (%)	31 (31.6)	31 (32.6)	38 (40.9)				
Graduate School, *n* (%)	9 (9.2)	19 (20.0)	5 (5.4)	0.005	0.313	0.033	0.003
English as primary language, *n* (%)	96 (98.0)	95 (100.0)	72 (77.4)	< 0.001	< 0.001	0.375	< 0.001
Insurance							
Private, *n* (%)	92 (93.9)	64 (67.4)	67 (72.0)	< 0.001	< 0.001	< 0.001	0.486
Medicaid, *n* (%)	0 (0.0)	6 (6.3)	16 (17.2)	< 0.001	< 0.001	0.011	0.020
Medicare, *n* (%)	5 (5.1)	23 (24.2)	8 (8.6)	< 0.001	0.337	< 0.001	0.004
Medicare and Medicaid, *n* (%)	1 (1.0)	1 (1.1)	2 (2.2)				
VA/CHAMPUS, *n* (%)	0 (0.0)	1 (1.1)	0 (0.0)				
Employment status							
Employed, *n* (%)	76 (77.6)	55 (57.9)	63 (67.7)	0.001	0.022	< 0.001	0.134
Unemployed, *n* (%)	10 (10.2)	7 (7.4)	15 (16.1)				
Unemployed due to MS, *n* (%)	2 (2.0)	27 (28.4)	9 (9.7)	< 0.001	0.201	< 0.001	0.001
SDOH composite indices							
ADI (state rank decile), mean (SD)	2.9 (2.3)	5.9 (7.7)	4.4 (2.6)	< 0.001	< 0.001	< 0.001	< 0.001
SVI, mean (SD)	35.5 (27.2)	59.2 (27.2)	46.4 (31.9)	< 0.001	0.022	< 0.001	0.006
EDSS							
Earliest evaluation, mean (SD)	1.3 (1.3)	2.1 (1.7)	2.0 (1.7)	< 0.001	0.001	< 0.001	0.504
FS 1—visual, mean (SD)	0.3 (0.8)	0.5 (1.1)	0.4 (0.8)	0.560	0.454	0.157	0.484
FS 2—brainstem, mean (SD)	0.2 (0.5)	0.4 (0.8)	0.4 (0.7)	0.143	0.007	0.022	0.729
FS 3—pyramidal, mean (SD)	0.5 (1.0)	1.1 (1.2)	0.9 (1.3)	0.002	0.017	< 0.001	0.111
FS 4—cerebellar, mean (SD)	0.5 (0.8)	0.9 (1.0)	0.8 (1.2)	0.010	0.154	< 0.001	0.089
FS 5—sensory, mean (SD)	0.4 (0.7)	0.5 (0.8)	0.5 (0.8)	0.200	0.136	0.031	0.602
FS 6—bowel/bladder, mean (SD)	0.1 (0.2)	0.4 (0.5)	0.2 (0.5)	0.005	0.006	< 0.001	0.032
FS 7—cerebral, mean (SD)	0.1 (0.3)	0.4 (0.6)	0.5 (0.6)	0.001	< 0.001	< 0.001	0.311
FS 8—ambulation, mean (SD)	0.2 (1.1)	0.8 (2.2)	0.7 (2.2)	0.227	0.042	0.010	0.570
Final evaluation, mean (SD)	1.7 (2.0)	2.8 (2.4)	2.6 (2.6)	0.003	0.020	0.001	0.421
FS 1—visual, mean (SD)	0.2 (0.4)	0.4 (1.1)	0.4 (0.9)	0.355	0.045	0.104	0.748
FS 2—brainstem, mean (SD)	0.2 (0.5)	0.4 (0.9)	0.4 (1.0)	0.198	0.014	0.014	0.995
FS 3—pyramidal, mean (SD)	0.8 (1.2)	1.2 (1.4)	1.5 (1.7)	0.027	0.006	0.049	0.336
FS 4—cerebellar, mean (SD)	0.4 (0.7)	1.0 (1.2)	0.9 (1.4)	0.003	0.018	< 0.001	0.242
FS 5—sensory, mean (SD)	0.5 (0.9)	0.6 (0.8)	0.6 (1.1)	0.691	0.566	0.312	0.687
FS 6—bowel/bladder, mean (SD)	0.1 (0.4)	0.4 (0.8)	0.5 (1.1)	0.050	0.015	0.001	0.378
FS 7—cerebral, mean (SD)	0.3 (0.5)	0.4 (0.6)	0.5 (0.9)	0.362	0.086	0.177	0.656
FS 8—ambulation, mean (SD)	1.0 (2.8)	2.0 (3.5)	2.1 (3.8)	0.024	0.014	0.001	0.512
EDSS ⩾ 4							
Earliest-recorded evaluation, *n* (%)	6 (6.1)	13 (13.7)	9 (9.7)	0.210	0.361	0.078	0.393
Last-recorded evaluation, *n* (%)	11 (11.2)	30 (31.6)	26 (28.0)	0.002	0.003	0.001	0.587
Change in EDSS from earliest to final, mean (SD)	0.4 (1.9)	0.6 (1.8)	0.6 (2.1)	0.812	0.716	0.506	0.808

SD: standard deviation; RRMS: relapsing-remitting multiple sclerosis, SPMS: secondary progressive multiple sclerosis, PPMS: primary progressive multiple sclerosis, DMT: disease-modifying therapy, BMI: body mass index, SDOH: social determinants of health, ADI: area deprivation index, SVI: social vulnerability index, EDSS: Expanded Disability Status Scale, FS: functional system.

The functional system subscores were also tabulated to better characterize disability. Black and Hispanic patients had higher brainstem, pyramidal, bowel/bladder, cerebral, and ambulation subscores compared to White patients at the earliest-recorded encounters. Black patients also had higher cerebellar and sensory subscores compared to White patients, and higher bowel/bladder subscores than either group. At the latest evaluations, Black and Hispanic patients had higher brainstem, pyramidal, cerebellar, bowel/bladder, and ambulation subscores compared to White patients. Hispanic patients also had higher visual subscores compared to White patients.

EDSS is an ordinal scale with unequal disability steps. A numeric increase of 0.5 or 1 in one part of the scale is not necessarily comparable to the same increase in another part of the scale. EDSS 4 is the lowest score at which disability is considered severe and is useful as a disability milestone.^23,30^ Similar proportions of patients with EDSS ⩾ 4 were observed at earlier visits, but at later visits, significantly more Black and Hispanic patients had reached or surpassed this milestone (*p* = 0.002).

There were no significant differences between groups in many clinical risk factors for MSAD including male sex, age at evaluation (either earliest- or last-recorded), age at first symptom onset, diagnostic lag, tobacco use, and years of DMT exposure. Patterns of DMT use among the groups were detailed in a previous publication.^20^ RRMS was the most common disease phenotype at the last-recorded encounter in all three groups. The Black patient group had the largest number of patients with PPMS, though overall numbers were low and the difference was only significant in comparison to the Hispanic patient group. Baseline vitamin D levels were lower among Hispanic patients compared to White patients and lower still among Black patients compared to Hispanic patients (*p* < 0.001), and the proportion of patients with vitamin D deficiency (⩽ 20 ng/mL) followed the same pattern (*p* < 0.001). A greater proportion of Black patients than White patients had a BMI ⩾ 30 (*p* = 0.034). Fewer Black patients carried a diagnosis of hypertension than either White or Hispanic patients (*p* = 0.010).

More pronounced differences were seen in the sociodemographic characteristics of the three groups. Fewer Hispanic patients reported completing at least 12 years of education (*p* = 0.028), whereas more Black patients had attended graduate school (*p* = 0.005). Black and Hispanic patients were both less likely to have private insurance (*p* < 0.001). The Black patient group included the largest proportion of Medicare patients (*p* < 0.001), and the Hispanic patient group included the largest proportion covered by Medicaid (*p* < 0.001). More Black and Hispanic patients than White patients reported being unemployed (*p* = 0.001). Markedly more Black patients (28.4% compared with 9.7% and 2.0% of Hispanic and White patients, respectively) reported being unemployed specifically because of MS (*p* < 0.001).

NLI measures were significantly higher (indicative of greater disadvantage) for neighborhoods of Black patients compared to those of Hispanic patients, which in turn had higher scores than those of White patients. This was true of both SVI (*p* < 0.001) and ADI (*p* < 0.001) in comparisons that included all three groups and was consistent among the three possible pairings.

Results of linear regression analysis are summarized in Table 3. All models were associated with *p*-values < 0.001. In the model that included only clinical characteristics, the coefficients for both Black race and Hispanic ethnicity were significantly associated with a higher EDSS score (*p* = 0.006 and *p* = 0.003, respectively). When individual-level SDOH indicators were included both associations were attenuated as reflected by smaller coefficients and larger *p*-values, and the association with Black race was no longer significant (*p* = 0.226 for Black race, *p* = 0.039 for Hispanic ethnicity). Neither Black race nor Hispanic ethnicity were significantly associated with changes in EDSS in either model that included variables for race and ethnicity and an SDOH NLI index. Variables that retained statistical significance in multivariable models included age at time of evaluation, PPMS phenotype, employment status, and the two NLI indices. The companion models that excluded race and ethnicity variables retained statistical significance, and there was minimal change in the adjusted *R*^2^ values compared to models that included race and ethnicity.

**Table 3.** Linear regression models of EDSS at last-recorded evaluation.

	Univariable models		Multivariable models											
	Coefficient (95% CI)	*p* Value	Clinical characteristics *p* < 0.001 Adjusted *R*^2^ = 0.150		Individual-level SDOH *p* < 0.001 Adjusted *R*^2^ = 0.209		Individual-level SDOH and ADI *p* < 0.001 Adjusted *R*^2^ = 0.211		Individual-level SDOH and ADI (race/ethnicity *excluded*) *p* < 0.001 Adjusted *R*^2^ = 0.209		Individual-level SDOH and SVI *p* < 0.001 Adjusted *R*^2^ = 0.202		Individual-level SDOH and SVI (race/ethnicity *excluded*) *p* < 0.001 Adjusted *R*^2^ = 0.198	
			Coefficient (95% CI)	*p* Value	Coefficient (95% CI)	*p* Value	Coefficient (95% CI)	*p* Value	Coefficient (95% CI)	*p* Value	Coefficient (95% CI)	*p* Value	Coefficient (95% CI)	*p* Value
Hispanic ethnicity	0.40 (−0.195, 1.000)	0.186	0.965 (0.341, 1.589)	0.003	0.688 (0.033, 1.342)	0.039	0.478 (−0.196, 1.152)	0.164	-	-	0.577 (−0.089, 1.244)	0.089	-	-
Black race	0.60 (0.004, 1.194)	0.049	0.902 (0.267, 1.538)	0.006	0.419 (−0.260, 1.098)	0.226	−0.001 (−0.741, 0.739)	0.999	-	-	0.185 (−0.527, 0.898)	0.609	-	-
Male sex	0.620 (−0.064, 1.304)	0.075	0.283 (−0.376, 0.942)	0.398	0.395 (−0.269, 1.059)	0.242	0.335 (−0.335, 1.004)	0.326	0.325 (−0.344, 0.995)	0.339	0.398 (−0.273, 1.068)	0.244	0.377 (−0.294, 1.049)	0.270
Age at final evaluation	0.057 (0.033, 0.080)	<0.001	0.041 (0.015, 0.067)	0.002	0.042 (0.015, 0.069)	0.002	0.044 (0.017, 0.072)	0.001	0.042 (0.015, 0.069)	0.002	0.042 (0.015, 0.069)	0.003	0.040 (0.013, 0.067)	0.004
Diagnostic lag	0.104 (0.022, 0.185)	0.013	0.073 (−0.005, 0.152)	0.067	0.077 (−0.003, 0.156)	0.058	0.076 (−0.004, 0.155)	0.061	0.078 (−0.001, 0.158)	0.053	0.077 (−0.003, 0.156)	0.058	0.079 (−0.001, 0.158)	0.053
PPMS phenotype	2.748 (1.548, 3.949)	<0.001	1.878 (0.646, 3.111)	0.003	1.673 (0.411, 2.935)	0.01	1.567 (0.272, 2.862)	0.018	1.495 (0.203, 2.786)	0.023	1.594 (0.295, 2.893)	0.016	1.543 (0.247, 2.839)	0.020
History of tobacco use	0.543 (−0.095, 1.182)	0.095	0.387 (−0.211, 0.985)	0.204	0.404 (−0.207, 1.016)	0.194	0.387 (−0.239, 1.013)	0.224	0.371 (−0.255, 0.997)	0.244	0.428 (−0.196, 1.051)	0.178	0.407 (−0.217, 1.031)	0.200
Hypertension	0.581 (−0.070, 1.231)	0.080	0.091 (−0.581, 0.764)	0.789	0.069 (−0.626, 0.765)	0.844	0.139 (−0.575, 0.853)	0.702	0.220 (−0.483, 0.923)	0.538	0.108 (−0.604, 0.820)	0.766	0.174 (−0.526, 0.875)	0.624
Diabetes Mellitus	1.425(0.105, 2.746)	0.035	0.934 (−0.318, 2.187)	0.143	1.255 (−0.034, 2.544)	0.056	1.070 (−0.264, 2.404)	0.115	1.128 (−0.205, 2.462)	0.097	1.102 (−0.235, 2.440)	0.106	1.153 (−0.185, 2.493)	0.091
Employed	−1.495 (−2.141, −0.848)	<0.001	-	-	−0.871 (−1.539, −0.203)	0.011	−0.774 (−1.461, −0.087)	0.027	−0.790 (−1.475, −0.105)	0.024	−0.856 (−1.541, −0.172)	0.014	−0.897 (−1.577, −0.216)	0.010
Private insurance	−1.327 (−1.989, −0.666)	<0.001	-	-	−0.800 (−1.535, −0.065)	0.033	−0.642 (−1.385, 0.101)	0.090	−0.680 (−1.414, 0.054)	0.069	−0.677 (−1.421, 0.067)	0.074	−0.751 (−1.485, −0.017)	0.045
ADI (state rank decile)	0.207 (0.108, 0.305)	<0.001	-	-	-	-	0.154 (0.045, 0.263)	0.006	0.152 (0.051, 0.253)	0.003	–	–	–	–
SVI (percentile rank)	1.488 (0.577, 2.398)	0.002	-	-	-	-	–	–	–	–	0.985 (0.047, 1.923)	0.040	1.018 (0.108, 1.927)	0.028

PPMS: primary progressive multiple sclerosis; SDOH: social determinants of health; ADI: area deprivation index; SVI: social vulnerability index; CI: confidence interval.

## Discussion

The striking lack of a significant association between Black race or Hispanic ethnicity and EDSS in models that include individual and neighborhood-level SDOH indicators sets our findings apart from previous studies. Marrie et al.^4^ reported that adjustment for income, insurance type, and education attenuated associations between Black race and poorer outcomes. Our analysis supplements these same indicators with ADI and SVI in an effort to more comprehensively study the role of SDOH. Gray-Roncal et al. performed a cross-sectional analysis of associations between neighborhood-level SDOH indicators and MS outcomes.^17^ Their analysis was performed separately for White and Black patients, whereas all groups in our sample were included in regression models so as to compare the strength of associations between race and ethnicity with EDSS and that of SDOH indicators. Abbatemarco et al.^18^ reported that higher ADI was associated with poorer outcomes and that Black patients represented a higher proportion of residents of the lowest ADI quartiles, but outcomes were not reported by race, and the role of ADI in racial disparities was not explored. None of these previous reports included a Hispanic patient subgroup. To our knowledge, this report is the first example of a longitudinal analysis including both individual and neighborhood-level SDOH indicators in White, Black, and Hispanic patients. Further investigation is required to establish a causative relationship between SDOH and MSAD disparities and clarify the mechanisms by which any causation occurs.

Clinical MSAD risk factors were mostly consistent across the three groups, and those differences that did exist are unlikely an adequate explanation for observed differences in MSAD. The larger proportion of PPMS among Black PwMS has been observed elsewhere though not consistently.^4,31^ The difference is notable, but the actual number of patients with PPMS in this study is not sufficient to explain the differences in EDSS. Vascular risk factors have been associated with MS disease course.^32^ Differences in these comorbidities observed in our study population did not suggest a uniform risk profile. For example, more Black patients had a BMI ⩾ 30 but fewer had hypertension. Differences in vitamin D level and deficiency were consistent with known differences among persons with darker skin tones, and the MS Sunshine Study suggested that these differences are not associated with MS risk.^33^

Sociodemographic characteristics differed among the three groups. English proficiency, education, and insurance provider all likely have ramifications for healthcare access over a patient’s lifespan, though a mechanistic analysis of such was not possible with the data available.

Though both Hispanic and Black patients had greater MSAD, there was a markedly higher number of Black patients who reported unemployment specifically attributable to MS. Further investigation could explore whether structural racism in the workplace, including discriminatory hiring and firing practices, might contribute to this difference. A greater number of Black patients attended graduate school. Even though a majority of this subgroup remained employed and had private insurance, having attended graduate school was not significantly related to EDSS score among Black patients (data not shown). This was a small subgroup with likely limited statistical power, but it can be hypothesized that these several advantages were not sufficient to overcome the deleterious influence of the larger scope of societal inequity.

The differences in neighborhood disadvantage as measured by SVI and ADI are consistent with patterns of residential segregation common in the United States.^10^ The indices represent a wide variety of social conditions, which appear relevant to MS disease course in the aggregate. Much remains to be discovered regarding the relative contributions of income, housing, transportation and other factors.

This study has important limitations. EDSS scores were calculated via retrospective review, and EDSS alone is unlikely to reflect all aspects of MS disease course. Uninsured patients are not represented in the sample being more commonly seen by other health systems in the Houston area, and the generalizability of the findings is therefore limited. Clinical encounters occurred at irregular intervals, and therefore sustained EDSS change could not be established by repeated assessment. This weakness is attenuated by the long follow-up period reflected in the data. Since change of address information was not available in the EMR at sufficiently regular time points, it is impossible to track the impact of change in NLI. A future study including relocation data might be recommended.

Ultimately, individual-level SDOH data are required to inform more definitive findings. That said, it would be interesting to investigate whether neighborhood conditions independently predict disease outcomes even when individual-level factors are accounted for.

Finally, adjusted *R*^2^ values indicate that less than a quarter of the variability in our data is explained by our models. Indeed, the full scope of the relationship between health and SDOH including structural racism is enormous,^8,10^ and considerable additional research is required to understand it comprehensively.

The importance of SDOH in racial and ethnic health disparities is broadly relevant to health and is uniquely illustrated by the history of MS epidemiology. MS was once thought to be rare among non-White persons.^34^ Evidence cited in support of this misconception was based on study samples likely not representative of the general population because of sampling bias caused by social factors and analyses that failed to account for differences in healthcare access.^2,35^ Failure to appreciate the relevance of SDOH to MS and other conditions has historically led to erroneous assumptions of biologic differences between races, and such assumptions have perpetuated structural inequity.^9,35^ Future investigations of differences in MS among racial and ethnic groups must appreciate structural inequities if they are to inform effective clinical practice and policy change.
